# Supplementary material for: Pathophysiological Significance of Neutrophilic Transfer RNA-Derived Small RNAs in Asymptomatic Moyamoya Disease
Source: Cells. 2021 May 1;10(5):1086. doi: 10.3390/cells10051086 (PMC8147334; doi:10.3390/cells10051086)
Supplement: Supplementary file 1 [file cells-10-01086-s001.zip › cells-1149161 3rd supplementary materials/Data Supplements/Data Supplement V.zip!/Data supplement 6-mRNAs targets of tsRNAs/tsRNA-Glu-CTC-003/Diagrams/tRF-Glu-CTC-003_vs_ENST00000262719(PHLPP1).pdf]

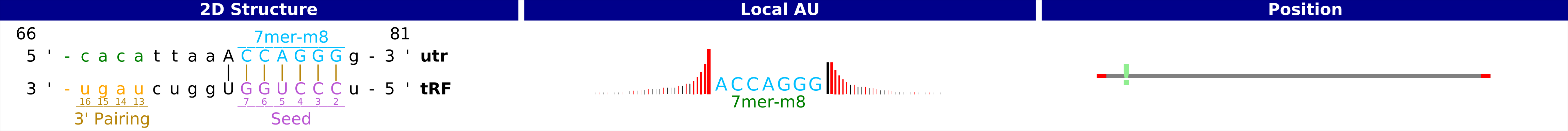
2D Structure
Local AU
Position
66
81
7mer-m8
5' - cacattaaACCAGGGg - 3' utr
3' - ugaucuggUGGUCCCu - 5' tRF
16 15 14 13
7 6 5 4 3 2
3' Pairing
Seed
ACCAGGG
7mer-m8
